# Loneliness around the world: Age, gender, and cultural differences in loneliness

**DOI:** 10.1016/j.paid.2020.110066

**Published:** 2021-02-01

**Authors:** Manuela Barreto, Christina Victor, Claudia Hammond, Alice Eccles, Matt T. Richins, Pamela Qualter

**Affiliations:** a Department of Psychology, University of Exeter, Exeter EX44QG, United Kingdom; b Department of Clinical Sciences, Brunel University London, UB8 3PH, United Kingdom; c Radio Science Unit, British Broadcasting Corporation (BBC), London W1A 1AA, United Kingdom; d Manchester Institute of Education, University of Manchester, M13 9PL, United Kingdom

**Keywords:** Loneliness, Age, Gender, Culture, BBC loneliness experiment

## Abstract

The BBC Loneliness Experiment provided a unique opportunity to examine differences in the experience of lonelines across cultures, age, and gender, and the interaction between these factors. Using those data, we analysed the frequency of loneliness reported by 46,054 participants aged 16–99 years, living across 237 countries, islands, and territories, representing the full range of individualism-collectivism cultures, as defined by Hofstede (1997). Findings showed that loneliness increased with individualism, decreased with age, and was greater in men than in women. We also found that age, gender, and culture interacted to predict loneliness, although those interactions did not qualify the main effects, and simply accentuated them. We found the most vulnerable to loneliness were younger men living in individualistic cultures*.*

## Highlights

• Younger people reported more loneliness than the middle-aged.
• The middle-aged reported more loneliness than older people.
• Men reported more loneliness than women.
• People in individualistic (vs. collectivist) countries reported more loneliness.
• Age, gender, and culture interacted to predict loneliness.

## 1 Introduction

Increasing attention is currently being paid to loneliness due to improved understanding of the impacts it has on individuals and communities (Jo Cox Commission, 2017; UK HM Government, 2018). Defined as the discrepancy between actual and desired social relationships (Peplau & Perlman, 1982), loneliness contributes negatively to well-being (Hawkley & Cacciopo, 2010; Holt-Lunstad & Smith, 2015), increasing use of health services (Christiansen et al., 2020), and impacting negatively on the economy by decreasing employee health, (Jeffrey, Abdallah, & Michaelson, 2017). Indeed, loneliness has been estimated to cost 2.5 billion per year to UK employers alone (Jeffrey et al., 2017). Understanding what might lead people to feel lonely and what keeps some people stuck in loneliness is crucial for the development of well targeted successful interventions to prevent and mitigate the effects of loneliness (Qualter et al., 2015).

The factors that influence the extent to which people feel lonely are, broadly speaking, those that affect desired or actual social relationships. Two people with the same objective number of close relationships might feel lonely to a different extent, if their desired relationships differ. At the same time, two people with the same desired number of close relationships might be lonely to a different extent if they do not feel their actual relationships are fulfilling. But what factors might affect what we want from social relationships?

Research in this area has examined differences in personality (e.g., extraversion), social skills (e.g., empathy, social anxiety), demographics (e.g., age), resources (e.g., time, money, availability of transport), physical mobility, among others (see British Red Cross and Co-Op, 2016; ONS, 2018; Pinquart & Sörensen, 2001, for reviews). The evidence for some of those factors is fairly well established, but is inconsistent for others. For example, it is clear that marriage or cohabitation protects against loneliness. However, the independent effects of age, gender, and culture remain unclear; exploration of how those individual differences might work intersectionally to predict loneliness is absent from the literature. That is because most studies are based on very small samples limiting analytical power and yielding unreliable results. Lack of diversity in samples precludes the examination of crucial interactions between demographic factors and where cultural differences have been examined, comparison is over a limited number of cultures.

In the current paper we use The BBC Loneliness Experiment dataset to examine the effects of culture, age, and gender on loneliness. This is a very large dataset drawn from the general population, with participants resident in 237 countries, islands, and territories, aged between 16 and 99 years, providing a unique opportunity to examine differences in the experience of lonelines across cultures, age, and gender, and the interaction between these factors.

### 1.1 Is loneliness affected by culture?

Cultures differ in the form and meaning of social behavior and ascribe different values and meaning to interpersonal relationships (Chen & French, 2008; Van Staden & Coetzee, 2010). Cultures are often classified as varying in levels of individualism and collectivism, but it remains unclear which of those types of cultures has a higher prevalence rate of loneliness. Cross-cultural comparisons of social relationships are most often made by reference to the concept of individualism vs. collectivism (Johnson & Mullins, 1987). Individualistic cultures place high value on self-reliance and are associated with loose social networks, primarily dominated by chosen relationships; collectivist cultures encourage interdependence and are patterned by tighter social networks, dominated by family and other ingroup members (Hofstede, 1997). While both types of culture involve risks to sociality, those tend to be linked to high social needs in collectivist societies and to low social contact in individualistic ones, both of which affect the match between ideal and actual relationships (Johnson & Mullins, 1987).

The majority of the evidence tends to suggest lower levels of loneliness in individualistic compared to collectivist countries (see Dykstra, 2009 for a review) although there is some evidence to the contrary (e,.g., Rokach, Orzeck, Cripps, Lackovic-Grgin, & Penezic, 2001; Van Tilburg, Havens, & de Jong Gierveld, 2002). Evidence from studies that measure individualism-collectivism at the individual (rather than country) level also reveal mixed findings (Heu, Van Zomeren, & Hansen, 2018; Jiang, Li, & Shypenka, 2018; Jylha & Jokela, 1990).

Those discrepancies are likely to be due, at least in part, to the theoretical and methodological complexity of cross-cultural comparisons, which lead to disputes about what qualifies as culture and introduces ambiguities in the interpretation of the data. Cultural differences are sometimes studied by comparing the responses of individuals with different cultural ancestry, but resident in the same country. However, that approach confounds culture with minority status, which can in itself affect loneliness (Doyle & Barreto, 2019; Madsen et al., 2016). A different approach is to compare the responses of individuals living in different countries. The problem, here, is that such comparisons usually involve very small samples of populations from a limited number of countries (sometimes only two) that are seen to represent a particular type of culture (such as individualist vs. collectivist), most often including only countries in Europe or North America (Chen et al., 2004; Stickley, Koyanagi, Koposov, Schwab-Stone, & Ruchkin, 2014). This can be contrasted with sampling a large number of participants across a wide number of countries that represent these cultural differences. Such an approach provides more confidence that findings are related to that particular cultural difference, rather than to the many other ways in which two countries might differ. Finally, the studies included different age groups, and it is very possible that culture affects loneliness differentially by age.

### 1.2 How might age affect loneliness?

It is a commonly held belief that loneliness is particularly prevalent among older people, but research does not support that proposition. One study reported no significant age differences in loneliness (Griffin, 2010). Other studies documented that younger respondents reported significantly more loneliness than older respondents (ONS, 2018; Schultz & Moore, 1988), with a linear decrease with age (ONS, 2018). And yet another set of studies has shown a U-shaped curve (of varying ‘flatness’), in which young adults and older people report more loneliness than those in middle age (Lasgaard, Friis, & Shevlin, 2016; Luhmann & Hawkley, 2016; Victor & Yang, 2012). Thus, there is some inconsistency in the pattern of effects across ontogeny. In addition, those studies include data from a small number of cultures, limiting our understanding of whether such age differences in loneliness are universal across the world.

Research focusing on the drivers of loneliness at the individual level can help clarify how loneliness might vary with age. It is now clear that loneliness is driven by a range of mechanisms that are partly developmental and partly socio-cultural (de Jong Gierveld, Tilburg, & Dykstra, 2006; Qualter et al., 2015). For example, adolescents and young adults are vulnerable to loneliness due to the instability of their social networks, related to changes in school, identity exploration, or physical changes that can make young people vulnerable to exclusion (Qualter et al., 2013, Qualter et al., 2015). Adolescence is fraught with tension between social connection (the need to belong) and individuation (Larson, Richards, Moneta, Holmbeck, & Duckett, 1996). Adolescents are expected to conform to the peer group and have intimate friendships, but they are also expected to develop independence from friends and family, which is seen as a central developmental task of the adolescent years. Many adolescents struggle in the quest to find a balance between those opposing expectations, which leads to loneliness (Qualter et al., 2015). The middle-aged might be particularly vulnerable to loneliness that is driven by work status, income, separation, or reduced availability of time due to work and caring responsibilities (Beeson, 2003; Leeflang, Klein-Hesselink, & Spruit, 1992; Luhmann & Hawkley, 2016; Rook, 2000). Loneliness among older people, in turn, often emerges due to loss of people in their social network (as a result of retirement or bereavement), living alone, or reduced mobility related to health conditions (Victor, Scambler, Bowling, & Bond, 2005). That is, different age groups are likely to experience specific challenges to social connection (Jopling & Sserwanja, 2016). Importantly, some of those might be universal, but others might be cultural, leading to different age patterns in samples drawn from different cultures (Yang & Victor, 2011). If we consider young adulthood as an example, the peer context is not of equal importance in different cultures (Liu, Li, Purwono, Chen, & French, 2015), suggesting that there may be different age-related patterns in loneliness across cultures that attach a different value to relationships with peers and community. In sum, if we consider the drivers of loneliness in different age groups, it becomes clear that some of those are cultural, raising questions about the interaction between age and culture on loneliness.

### 1.3 Does gender affect loneliness?

As with age, gender differences in loneliness are often assumed, and sometimes the evidence suggests that women report more loneliness than men, irrespective of age (Pinquart & Sörensen, 2001; see also Nikolaisen & Thorsen, 2014; ONS, 2018). Again, considering the drivers of loneliness among men and women might clarify how gender affects loneliness in different age groups and cultures. Although women are socialized to develop a larger and more active social network, potentially protecting them from loneliness (Okun & Keith, 1998), it is also clear that women tend to live longer than men and are, therefore, more likely to be affected by widowhood, or likely to assume the role of care-giver for their spouse. As such, it is possible that women are more lonely than men, especially in old age, suggesting that it is important to examine the effect of gender on loneliness across different age groups and cultures. While a recent meta-analysis did not support the idea of gender differences in loneliness (Maes, Qualter, Vanhalst, Van den Noortgate, & Goossens, 2019), with no effects of either age or culture, few studies in that meta-analyses examined a number of cultures.

Where gender differences are found in loneliness, it might also reflect differences in the extent to which men and women are willing to report loneliness. Indeed, research has shown that men are more reluctant than women to admit feeling lonely (Borys & Perlman, 1985) and that men who feel lonely are more stigmatized than women who express the same feeling (Lau & Gruen, 1992). Stigma is, however, culturally-specific, raising the possibility that gender differences in self-reports of loneliness might vary across cultures, and may be more evident when a number of cultures are examined. Likewise, since loneliness is commonly associated with old age, it might also appear less stigmatizing to admit to loneliness in old age, suggesting a possible interplay between gender, age, and culture.

### 1.4 The present research

In the current paper, we use data from the BBC Loneliness Experiment to examine the effects of age, gender, and culture in a much larger and diverse sample than has been done before. Crucially, given the magnitude and demographic diversity of our sample, we are also able to examine the interplay between those factors. Participants indicated the extent to which they experienced loneliness frequently, intensely, and for how long (see Qualter, Barreto, Petersen, & Victor, 2020). The results are similar for loneliness frequency, intensity, and duration. To facilitate comparisons with prior research using the UCLA scale, we report results for frequency in the text and provide the results for the intensity and duration of loneliness in the supplementary materials.

## 2 Method

Participants took part in an online survey launched on BBC Radio 4 and BBC World Service, and mentioned in several other media that picked up on the event. Participants who were interested could access the study online and were first provided with information about the study. Those who agreed to participate answered a range of questions about their social life and their experiences with loneliness, only a small portion of which are the focus of this paper. A total of 54,988 people completed the survey. In this paper we report data from the 46,054 participants who had provided data on the variables of interest.

The analyses reported in this paper follow a quasi-experimental design, with age, gender, and culture as quasi-experimental between participant factors. A total of 47,381 participants indicated their age by entering it in a free text box. For gender, 49,019 participants indicated whether they were male, female, other, or whether they preferred not to answer. Gender was an independent variable (IV) of interest in this paper, but only participants who indicated their gender as male or female were included in the analyses; we did not have sufficient data to perform a meaningful analysis of the ‘other’ category. To operationalize individualism, participants were asked their country of residence with a total of 48,411 participants providing this information. Then, each participant was assigned a score on the Hofstede's Individualism Index based on their country of residence (1997). This includes scores for 101 countries on a 100-point scale ranging from 6 (Guatemala) to 91 (United States), with higher scores representing higher individualism. A total of 497 participants were excluded because their country of residence was not included in Hofstede's database. This led to a sample of 46,054 participants who were either male or female, provided information about their age and place of residence, and could be classified according to the Hofstede's index. Demographics of participants whose data are used in the current paper are described in Table 1.

**Table 1:** Characteristics of final sample used in the current study.

Male %	32.3%
Mean age in years (SD)	49.7 (15.44)
Age range in years	16–99
Residing in UK^a^	34239
Mean Hofstede Indivisualism Index (SD)	83.74 (14.99)
% falling below 43 on Hofstede Individualism Index	4.8%
In full-time work %^b^	45%
In part-time work %^b^	17.6%
In upaid work %^b^	3.4%
Student (full or part-time) %^b^	6%
Retired %^b^	23%
Unemployed %^b^	5.6%
Agreed that financial resources met their needs fairly well or very well %	83.3%
Single %	29.1%
Married or in civil partnership%	31.1%
In a relationship, but not cohabiting%	5.7%
Cohabiting %	9%
Separated or divorced %	19%
Widowed %	6.2%
Lives alone	40.6%

Notes:

a Largest sample in any residing country;

b Percentages do not add to 100% due to missing responses.

Loneliness was measured by asking participants to answer questions from the UCLA Loneliness Scale (Russell, 1996): Do you feel a lack of companionship?, Do you feel left out?, Do you feel isolated from others?, and Do you feel in tune with people around you? Due to space restrictions, we used the validated four item version of this scale, so as to allow particiants to rate each item on frequency, intensity, and duration. Participants indicated, for each question, how often that happened to them on a sliding scale from 1 (never) to 5 (always). The scale, averaged across the four ratings, was reliable (α = 0.84).

Ethical approval was obtained for this study prior to data collection from the University Research Ethics Committee at XXXX. The study followed ethical guidelines by the British Psychological Society and the Declaration of Helsinki (2013). Data collection took place between February and May 2018. The study took approximately 45 mins to complete. Those who participated did so in a voluntary capacity.

## 3 Results^4^

Table 2 provides the correlations between all variables. To examine how age, gender, and individualism-collectivism, and their interactions, predicted loneliness, we conducted separate hierarchical regression analyses, with the main effects in step 1 and the two and three way interactions in step 2. Gender was dummy coded as −1 = male and + 1 = female and both age and individualism scores were standardized (zscores) for the regression analyses. To probe the significant interactions, we used model 1 of PROCESS (Hayes, 2017) and plotted the slopes ±1SD above and below the mean (for individualism one SD above the mean was replaced with the maximum value because it was outside the range of the data), including all other main effects and interactions as covariates in the model.

**Table 2:** Unstandardized means (SDs) and intercorrelations (and subsample sizes) between all variables.

	*M (SD)*	Loneliness Frequency	Individualism	Age
Loneliness frequency	2.64 (1.12)			
Individualism	83.78 (14.95)	0.03 p < .001 (43453)		
Age	49.70 (15.44)	−0.11 *p* < .001 (43453)	0.16 *p* < .001 (46054)	
Gender	67.7% female	−0.07 p < .001 (43453)	0.10 p < .001 (46054)	0.01 *p* < .010 (46054)

Notes: Samples for analyses vary because not all participants provided responses to all measures.

Gender was coded −1 = men, +1 = women.

All independent variables emerged as significant predictors of loneliness frequency. Specifically, age was negatively associated with loneliness (β = −0.12, *t* = −24.81, *p* < .001, 95% CI [−0.145, −0.124]), with older people reporting less frequent loneliness than younger people. Gender was also negatively associated with loneliness (β = −0.08, *t* = −15.73, *p* < .001, 95% CI [−0.102, −0.076]), with men reporting more frequent loneliness than women. Individualism was positively associated with loneliness (β = 0.06, *t* = 12.04, *p* < .001, 95% CI [0.055, 0.076], with people living in more individualistic societies reporting more frequent loneliness than people living in more collectivistic societies. The results also revealed significant interactions between Age X Individualism (β = 0.03, *t* = 4.66, *p* < .001, 95% CI [0.015, 0.036]), Gender X Individualism (β = −0.02, *t* = −3.47, *p* < .001, 95% CI [−0.033, −0.009]), and Age X Gender (β = 0.01, *t* = 2.13, *p* = .033, 95% CI [0.001, 0.024]), but no significant three-way interaction between those predictors (β = −0.004, *t* = −0.78, *p* = .433, 95% CI [−0.015, 0.006]). The inclusion of the interaction terms increased the predictive power of the model from R^2^ = 0.020, *F*(3, 43,449) = 299.78, *p* < .001 to R^2^ = 0.021, Δ R^2^ = 0.001, *F*(4, 43,445) = 10.24, *p* < .001.

**Fig. 1:**
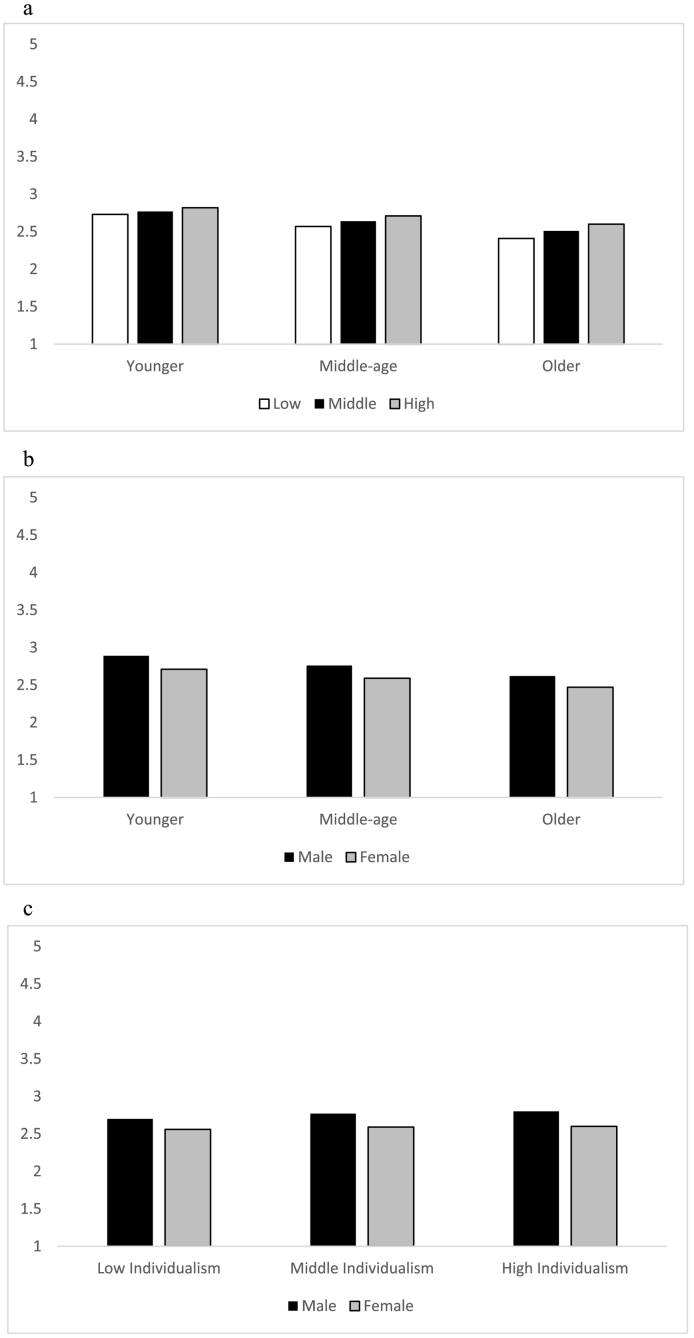
Unstandardized Loneliness Frequency as a function of Age and Individualism (1a), Age and Gender (1b), and Gender and Individualism (1c).

Regarding the interaction between Age and Individualism (see Fig. 1a), the results show that loneliness increased as individualism increased, irrespective of age, but this effect was stronger for older than younger participants (β_younger_ = 0.06, β_middle-age_ = 0.08; β_older_ = 0.11, all *p*s < 0.001). There was also a steady reduction in reported loneliness as age increased, irrespective of cultural group, although somewhat stronger for participants living in collectivist nations (β_collectivist_ = −0.16; β_middle_ = −0.14; β_individualistic_ = −0.13, all *p*s < 0.001).

For the interaction between Age and Gender (see Fig. 1b), we see that loneliness decreases with age for both male and female participants, although the effect of age is slightly stronger for males than females (β_males_ = −0.15; β_females_ = −0.13, *p*s < 0.001). In turn, male participants reported more loneliness than female participants at all ages, but this effect of gender was weaker for older than younger or middle aged participants (β_younger_ = −0.10; β_middle-age_ = −0.09; β_older_ = −0.08, all *p*s < 0.001).

With regard to the interaction between Gender and Individualism (see Fig. 1c), the results show that individualism was associated with more frequent loneliness for both male and female participants, but that effect of culture was stronger for males than females (β_males_ = 0.10; β_females_ = 0.06, *p*s < 0.001). In turn, male participants reported more frequent loneliness than female participants across all levels of individualism, with this gender effect being somewhat stronger for participants living in more individualistic nations (β_collectivist_ = −0.07; β_middle_ = −0.09; β_individualistic_ = −0.10, all *p*s < 0.001).

## 4 Discussion

We analysed the frequency of loneliness reported by 46,054 participants aged 16–99 years, living across 237 countries, islands, and territories, representing the full range of individualism-collectivism cultures, as defined by Hofstede (1997). We found that loneliness increased with individualism, decreased with age, and was greater in men than in women. We also found that age, gender, and culture interacted to predict loneliness. However, those interactions did not qualify the main effects, they simply accentuated them. We found the most vulnerable to loneliness were younger men living in individualistic cultures.

The age pattern identified in this study is consistent with the results of the recent ONS (2018) report, which used data from a representative sample, but was restricted to the UK. Because our data are cross-sectional, this pattern may not reflect developmental processes, but, instead, historical factors that differentially affected younger and older respondents, such as less stigma associated with (reporting) loneliness when younger (vs older) participants were growing up. Nevertheless, our results add to the evidence that loneliness is not unique to older people and might characterise the young rather than older age groups.

The finding that loneliness was higher in individualistic compared to collectivist cultures should also be interpreted with caution. The vast majority of participants resided in individualistic countries, particularly the UK, where loneliness has been a focus of discussion in the media. However, that does not explain why the effect was stronger for men and younger people, with individualistic cultures being particularly isolating for younger men (or collectivist cultures being particularly beneficial for older women). We propose that there is something about individualism that enhances loneliness, particularly if other risk factors (e.g., young age) are present.

We found that loneliness was higher among men than among women, which is contrary to the findings from the ONS (2018) survey. That might mean men need particular conditions to speak about loneliness, which our on-line survey may have afforded. In fact, our male participants were no more reluctant than female participants to admit feeling lonely, giving us confidence that the results reflect genuine feelings. That might have been facilitated by the fact participants were explicitly invited to join others (the radio host, the research team, other listeners) to reflect on loneliness, making it easier to do so, particularly for men. That, however, underlines the fact that this sample is not representative of the broader population—only, potentially, of those who are willing to express their feelings of loneliness. It is, however, important to note that even though the sample is not representative of the populations of the respective countries, contrary to prior research, the 237 countries sampled are representative of the full spectrum of individualistic versus collectivistic cultures.

In some of our other work, we found it useful to differentiate between loneliness frequency, intensity, and duration (Qualter et al., 2020), but those dimensions of loneliness were not differentially predicted by age, gender, or culture in this study (see supplementary materials). That is, young men living in individualistic cultures were more vulnerable not only to frequent loneliness, but also to loneliness that was more intense and longer lasting.

No causal inferences can be made on the basis of this cross-sectional data. As already indicated, it must also be kept in mind that we used a large, but non-representative sample of participants who volunteered to speak about their feelings of loneliness that, importantly, complements and extends other studies that used more rigorous sampling procedures (ONS, 2018) Finally, it is important to acknowledge that the effects we found were very small, although consistent across all three loneliness dimensions. We take this to mean that those effects are real and that loneliness is a fairly universal experience across demographic categories. Data are also provided by a large sample of individuals of different ages and from a large number of different countries, provided statistical power to fully explore effects. Thus, findings provide new insights about how culture moderates the effects of age and gender to predict experiences of loneliness. They, thus, are of importance for those who wish to develop public policy surrounding loneliness and/or design interventions for loneliness.

## CRediT authorship contribution statement

**Manuela Barreto:**Conceptualization, Methodology, Funding acquisition, Resources, Investigation, Formal analysis, Writing - original draft, Writing - review & editing.**Christina Victor:**Conceptualization, Methodology, Funding acquisition, Resources, Investigation, Formal analysis, Writing - review & editing.**Claudia Hammond:**Conceptualization, Methodology, Funding acquisition, Resources, Investigation, Writing - review & editing.**Alice Eccles:**Resources, Investigation, Data curation, Writing - review & editing.**Matt T. Richins:**Resources, Investigation, Data curation, Writing - review & editing.**Pamela Qualter:**Conceptualization, Methodology, Funding acquisition, Resources, Project administration, Investigation, Formal analysis, Writing - review & editing.

## Declaration of competing interest

None.

## Appendix A Supplementary data

Supplementary material Image 1

## Footnotes

4 Additional analyses were conducted to examine whether considering individualism on the basis of participant's birth country (instead of on the basis of their country of residence) would yield the same results as reported above. Overall, the results obtained when considering birth place were similar, but effects somewhat weaker, than when considering place of residence.
