# Supplementary material for: Loneliness around the world: Age, gender, and cultural differences in loneliness
Source: Pers Individ Dif. 2021 Feb 1;169:110066. doi: 10.1016/j.paid.2020.110066 (PMC7768187; doi:10.1016/j.paid.2020.110066)
Supplement: Supplementary file 1 — Supplementary material [file mmc1.docx]

**SUPLEMENTARY MATERIALS**

**Loneliness intensity**

All independent variables emerged as significant predictors of loneliness intensity. Specifically, age was negatively associated with loneliness intensity (β = -.15, *t* = -30.29, *p* < .001, 95% CI [-.177, -.155]), with older people reporting less loneliness intensity than younger people. Gender was also negatively associated with loneliness intensity (β = -.03, *t* = -6.64, *p* < .001, 95% CI [-.050, -.027]), with men reporting more loneliness intensity than women. Individualism was positively associated with loneliness intensity (β = .06, *t* = 13.04, *p* < .001, 95% CI [.061, .083]), with people living in more individualistic societies reporting more intense loneliness than people living in more collectivistic societies. The results also revealed significant interactions between Age X Individualism (β = .03, *t* = 4.93, *p* <.001, 95% CI [.016, .038]), Gender X Individualism (β = -.02, *t* = -2.97, *p* =.003, 95% CI [-.031, -.006]), and Age X Gender (β = .01, *t* = 2.82, *p* = .022, 95% CI [.002, .025]), but (as with loneliness frequency) no significant three-way interaction between these predictors (β = -.006, *t* = -1.14, *p* = .254, 95% CI [-.017, .005]). The inclusion of the interaction terms increased the predictive power of the model from R^2^=.023, *F*(3, 42142) = 338.66, *p* < .001 to R^2^ = .024, ΔR^2^= .001, *F*(4, 42138) = 10.08, *p* <.001.

Regarding the interaction between Age and Individualism (see Figure 2a), the results show that reported loneliness intensity increased as individualism increased, irrespective of age, but this effect was stronger for older than younger participants (β_younger_ = .06, β_middle-age_ = .09; β_older_ = .11, all *p*s < .001). There was also a steady reduction in reported loneliness intensity as age increased, irrespective of cultural group, though somewhat stronger for participants living in more collectivist nations (β_collectivist_= -.20; β_middle_ = -.17; β_individualistic_ = -.16).

For the interaction between Age and Gender (see Figure 2b), we see that reported loneliness intensity decreases with age for both male and female participants, though this effect of age is slightly stronger for males than females (β_males_= -.18; β_females_ = -.16, *p*s < .001). In turn, male participants reported greater loneliness intensity than female participants at all ages, but this effect of gender was weaker for older than younger or middle aged participants (β_younger_= -.05; β_middle-age_ = -.04; β_older_ = -.03, all *p*s < .001).

With regard to the interaction between Gender and Individualism (see Figure 2c), the results show that individualism was associated with greater reported loneliness intensity for both male and female participants, but this effect of culture was stronger for males than females (β_males_= .10; β_females_ = .07, *p*s < .001). In turn, male participants reported greater loneliness intensity than female participants across all levels of individualism, with this gender effect being somewhat stronger for participants living in more individualistic nations (β_collectivist_= -.02; β_middle_ = -.04; β_individualistic_ = -.05, all *p*s < .002).

Figure 2. Loneliness Intensity as a function of Age and Individualism (2a), Age and Gender (2b), and Gender and Individualism (2c)

(2a)

(2b)

(2c)

**Loneliness duration**

Again, all independent variables emerged as significant predictors of the duration of loneliness episodes. Specifically, age was negatively associated with loneliness duration (β = -.08, *t* = -15.55, *p* < .001, 95% CI [-.135, -.105]), with older people reporting less lasting loneliness than younger people. Gender was also negatively associated with the duration of loneliness episodes (β = -.07, *t* = -14.02, *p* < .001, 95% CI [-.130, -.098]), with men reporting longer lasting loneliness than women. Individualism-collectivism was positively associated with loneliness duration (β = .05, *t* = 9.31, *p* < .001, 95% CI [.058, .088], with people living in more individualistic societies reporting longer lasting loneliness than people living in more collectivistic societies. The results also revealed significant interactions between Age X Individualism (β = .03, *t* = 4.38, *p* <.001, 95% CI [.019, .050]), Gender X Individualism (β = -.02, *t* = -2.87, *p* =.004, 95% CI [-.042, -.008]). For this measure, neither the interaction between Age X Gender (β = .003, *t* = .54, *p* = .587, 95% CI [-.012, .021]), nor the three-way interaction between all predictors (β = -.004, *t* = -.74, *p* = .462, 95% CI [-.021, .010]) were significant. The inclusion of the interaction terms increased the predictive power of the model from R^2^=.013, *F*(3, 42142) = 36054, *p* < .001 to R^2^ = .014, ΔR^2^= .001, *F*(4, 36050) = 7.20, *p* <.001.

Regarding the interaction between Age and Individualism (see Figure 3a), the results show that reported duration of loneliness episodes increased as individualism increased, irrespective of age, but this effect was stronger for older than younger participants (β_younger_ = .06, β_middle-age_ = .09; β_older_ = .13, all *p*s < .001). There was also a steady reduction in the reported duration of loneliness as age increased, irrespective of cultural group, though somewhat stronger for participants living in more collectivist nations (β_collectivist_= -.15; β_middle_ = -.12; β_individualistic_ = -.11).

For the interaction between Age and Gender (see Figure 3b), we see that the reported duration of loneliness episodes decreases with age for both male and female participants, though this effect of age is very slightly stronger for males than females (β_males_= -.13; β_females_ = -.12, *p*s < .001). In turn, male participants reported greater loneliness frequency than female participants at all ages, with the effect being very slightly stronger for younger participants (β_younger_= -.12; β_middle-age_ = -.11; β_older_ = -.11, all *p*s < .001).

With regard to the interaction between Gender and Individualism (see Figure 3c), the results show that individualism was associated with a greater reported duration of loneliness episodes for both male and female participants, but this effect of culture was stronger for males than females (β_males_= .11; β_females_ = .07, *p*s < .001). In turn, male participants reported longer duration of loneliness episodes than female participants across all levels of individualism, with this gender effect being somewhat stronger for participants living in more individualistic nations (β_collectivist_= -.09; β_middle_ = -.12; β_individualistic_ = -.13, all *p*s < .001).

Figure 3. Loneliness Duration as a function of Age and Individualism (3a), Age and Gender (3b), and Gender and Individualism (3c)

(3a)

(3b)

(3c)
